# Antiseizure medication withdrawal risk estimation and recommendations: A survey of American Academy of Neurology and EpiCARE members

**DOI:** 10.1002/epi4.12696

**Published:** 2023-02-14

**Authors:** Samuel W. Terman, Renate van Griethuysen, Carole E. Rheaume, Geertruida Slinger, Anisa S. Haque, Shawna N. Smith, Wesley T. Kerr, Charlotte van Asch, Willem M. Otte, Carolina Ferreira‐Atuesta, Marian Galovic, James F. Burke, Kees P. J. Braun

**Affiliations:** ^1^ Department of Neurology University of Michigan Ann Arbor Michigan USA; ^2^ Department of Clinical Neurophysiology and Sleep Centre SEIN Zwolle The Netherlands; ^3^ American Academy of Neurology Minneapolis Minnesota USA; ^4^ Department of Child Neurology UMC Utrecht Brain Center, University Medical Center Utrecht, member of ERN EpiCARE Utrecht University Utrecht The Netherlands; ^5^ University of Michigan Medical School Ann Arbor Michigan USA; ^6^ Department of Health Management and Policy University of Michigan School of Public Health Ann Arbor Michigan USA; ^7^ Department of Clinical and Experimental Epilepsy (DCEE) NIHR University College London Hospitals Biomedical Research Centre UCL Queen Square Institute of Neurology London UK; ^8^ Chalfont Centre for Epilepsy Chalfont St Peter UK; ^9^ Department of Neurology The Icahn School of Medicine at Mount Sinai New York New York USA; ^10^ Department of Neurology, Clinical Neuroscience Center University Hospital and University of Zurich Zurich Switzerland; ^11^ MRI Unit Chalfont Centre for Epilepsy Chalfont St Peter UK; ^12^ Department of Neurology Ohio State University Columbus Ohio USA

**Keywords:** antiseizure medications, discontinuation, epilepsy, survey

## Abstract

**Objective:**

Choosing candidates for antiseizure medication (ASM) withdrawal in well‐controlled epilepsy is challenging. We evaluated (a) the correlation between neurologists' seizure risk estimation (“clinician predictions”) vs calculated predictions, (b) how viewing calculated predictions influenced recommendations, and (c) barriers to using risk calculation.

**Methods:**

We asked US and European neurologists to predict 2‐year seizure risk after ASM withdrawal for hypothetical vignettes. We compared ASM withdrawal recommendations before vs after viewing calculated predictions, using generalized linear models.

**Results:**

Three‐hundred and forty‐six neurologists responded. There was moderate correlation between clinician and calculated predictions (Spearman coefficient 0.42). Clinician predictions varied widely, for example, predictions ranged 5%‐100% for a 2‐year seizure‐free adult without epileptiform abnormalities. Mean clinician predictions exceeded calculated predictions for vignettes with epileptiform abnormalities (eg, childhood absence epilepsy: clinician 65%, 95% confidence interval [CI] 57%‐74%; calculated 46%) and surgical vignettes (eg, focal cortical dysplasia 6‐month seizure‐free mean clinician 56%, 95% CI 52%‐60%; calculated 28%). Clinicians overestimated the influence of epileptiform EEG findings on withdrawal risk (26%, 95% CI 24%‐28%) compared with calculators (14%, 95% 13%‐14%). Viewing calculated predictions slightly reduced willingness to withdraw (−0.8/10 change, 95% CI −1.0 to −0.7), particularly for vignettes without epileptiform abnormalities. The greatest barrier to calculator use was doubting its accuracy (44%).

**Significance:**

Clinicians overestimated the influence of abnormal EEGs particularly for low‐risk patients and overestimated risk and the influence of seizure‐free duration for surgical patients, compared with calculators. These data may question widespread ordering of EEGs or time‐based seizure‐free thresholds for surgical patients. Viewing calculated predictions reduced willingness to withdraw particularly without epileptiform abnormalities.

**Key Points**

- We performed an international survey of neurologists regarding antiseizure medication (ASM) withdrawal decisions.
- There was wide variation in risk estimation and recommendations in response to identical vignettes.
- Clinicians tended to overpredict risk for vignettes with abnormal EEGs and postsurgical cases and underpredict risk for vignettes continuing ASMs with normal EEGs, compared with calculators.
- Viewing calculator results tended to slightly reduce the chance of recommending ASM withdrawal, particularly for vignettes with a normal EEG.
- The greatest barrier to using the currently available risk calculator was being unsure regarding its accuracy.

## 1 INTRODUCTION

Two‐thirds of patients with epilepsy become seizure‐free on antiseizure medications (ASMs).^1^ For these patients, a key question is whether ASMs are necessary indefinitely. ASMs reduce morbidity and improve quality of life by decreasing seizures.^2^, ^3^ However, adverse effects reduce quality of life,^4^, ^5^, ^6^, ^7^ and risk declines with longer seizure‐freedom.^8^, ^9^ Thus, guidelines have endorsed considering withdrawal after detailed counseling.^10^, ^11^, ^12^

Accurate postwithdrawal seizure risk is key to optimizing decision‐making. Physicians often overestimate treatment benefits and underestimate harms, and risk calculators tend to outperform humans at estimating risk.^13^ Individualized postwithdrawal seizure risk calculators exist for medical^14^, ^15^ and surgical^16^ patients. Yet, the degree to which clinicians' intuitive estimate of postwithdrawal seizure risk (“clinician predictions”) align with model (“calculated”) predictions remains unclear, which may inform when viewing calculated results might most useful. Furthermore, recent guidelines highlight the need for data evaluating the influence of post‐ASM withdrawal prediction tools on recommendations.^12^ One study found that viewing calculated predictions decreased willingness to withdraw ASMs.^17^ However, that study included a small single‐center sample, did not include children or postsurgical cases, and was based on a now‐outdated calculator, thus applicability to current practice is unclear. Another survey documented variability in clinicians' ASM withdrawal recommendations but did not assess clinicians' risk predictions or the influence of viewing calculator results.^18^

To better understand current physician practice patterns surrounding ASM withdrawal decisions, we conducted an international survey of neurologists. We evaluated (a) the correlation between clinician predictions and calculated predictions, (b) how viewing calculated predictions influences ASM withdrawal recommendations, and (c) barriers to using risk calculation.

## 2 METHODS

### 2.1 Participants

We recruited neurologists. This included epileptologists and nonepileptologists, to compare responses, and given many nonepileptologists care for epilepsy patients.^19^ The first group included current US AAN members. Invitees were ≤68 years old, practicing in general neurology or with a subspecialty of epilepsy or clinical neurophysiology, and had not responded to another AAN survey in the previous 6 months. This left 5649 US AAN members; we randomly sampled 4001 (to leave participants for other AAN survey priorities). The second group consisted of all 403 eligible European AAN members, and a third non‐AAN group included 519 EpiCARE members. EpiCARE is a European network treating complex epilepsies, spanning 28 institutions across 24 countries. We asked respondents to disregard duplicate invitations if they were European AAN and EpiCARE members. We kept only the first attempt for 13 respondents who began the survey twice. Individuals received up to three email reminders, with recruitment spanning 5 months (6/1/2021‐October 31, 2021). There was no compensation.

### 2.2 Procedures involving human subjects

This study was approved by the University of Michigan IRB. The first page of the survey requested informed consent.

### 2.3 Survey variables and design

We assembled experts in ASM withdrawal decisions and developed a cross‐sectional survey collaborating with the American Academy of Neurology (AAN) (Appendix S1). The research team met to discuss objectives and drafted content to address objectives. All members (including an implementation scientist, epileptologists, nonepileptologist neurologists, statisticians, and a research coordinator) pretested the survey, and questions were refined until all members agreed that questions were clear and met objectives. The survey was designed in English, programmed into Qualtrics, and distributed electronically.

To evaluate for nonresponse bias, we compared demographics of all invited AAN members stratified by whether they consented to take the survey. These data included age, sex, race, geographic region, academic vs nonacademic practice, percent of time spent in research, and subspecialty. This was possible only for AAN members, because AAN's member database contains demographic information for all members, whereas EpiCARE data were available only from respondents.

We asked respondents whether they treat mostly adults or children, what percent of their patient practice is spent evaluating seizures or patients with epilepsy surgery, whether they are board‐certified in epilepsy or clinical neurophysiology, and their years of experience treating epilepsy patients.

While it is impossible to design vignettes presenting the full complexity of clinical care, we developed specific vignettes intended to represent a broad range of representative cases (Table S1). Vignettes spanned children and adults, surgical and nonsurgical (“medical”) cases, different epilepsy etiologies, and different durations of seizure‐freedom. Respondents were randomized to complete half of the clinical scenarios for which they were eligible, based on whether they treat adults vs children, and any postsurgical patients. Respondents viewed medical then surgical vignettes, if they treat both populations. We did not present pediatric surgical vignettes, given a previous overlapping study.^20^

Each vignette began with a reported normal EEG (we did not show actual EEG images). We asked respondents to estimate the patient's risk of having another seizure in the next 2 years (“clinician prediction”) if the patient withdrew vs continued ASMs. We did not specify any precise withdrawal schedule or duration, given no significance between faster vs slower tapering.^12^, ^21^ We also asked how likely the respondent would be to recommend withdrawal on a Likert scale (0/10: extremely unlikely; 5/10: neither likely nor unlikely; 10/10: extremely likely) under different scenarios such as whether the patient's job required driving, whether they were experiencing ASM‐related side effects, or wished for future pregnancy. Then, we repeated these questions in the presence of interictal epileptiform EEG abnormalities.

For medical vignettes, we then showed respondents calculated predictions of 2‐year seizure relapse risk if the patient withdrew vs continued ASMs and requested an updated recommendation regarding how likely they would be to advise withdrawal. We obtained postwithdrawal calculated predictions from Lamberink and colleagues' online risk calculator (http://epilepsypredictiontools.info/aedwithdrawal). This calculator was developed from 1769 patients pooling 10 real‐world datasets, demonstrated moderate performance during internal‐external cross‐validation (area under the curve 0.65), and provides the most rigorous currently available individualized postwithdrawal seizure risk prediction.^14^ To obtain “continuation” calculated predictions of 2‐year seizure relapse, we multiplied postwithdrawal calculated predictions by 50%, as informed by two randomized trials. (a) The largest randomized trial to date found 41% vs 22% relapse by 2 years.^22^ (b) The other trial relevant to adults found a 1‐year relapse risk of 15% vs 7% during double‐blinded follow‐up.^23^ No literature currently enables further individualized relative risk reductions.

For surgical vignettes, we similarly obtained clinician predictions and withdrawal recommendations. However, while a surgical postwithdrawal online risk calculator has very recently been validated (Ferreira‐Atuesta and colleagues^16^; https://predictepilepsy.github.io/), its validation was not yet complete at the time of this survey. Thus, we incorporated surgical calculated predictions into our analysis but not the actual survey. We also did not incorporate postsurgical continuation calculated risks, in the absence of RCT data.

### 2.4 Statistical analysis

We compared clinician vs calculated predictions using scatterplots and Spearman's correlation coefficients with 1000 bootstrapped replications to obtain confidence intervals [CI]. We also displayed violin plots of clinician vs calculated predictions by vignette. Because the Lamberink model overpredicted risk in two external validation studies,^24^, ^25^ and a third validation study demonstrated poor calibration,^26^ we performed a sensitivity analysis using external predictions. We chose the predicted risk from the Lamberink model that corresponded to the published logistic calibration curve from Lin and colleagues (their Figure 4A).^24^ To compute mean clinician predictions with 95% CI's for each vignette and thus to compare mean clinician vs calculated predictions, we then performed generalized linear models.^27^ The outcome consisted of clinician predictions (0%‐100%) for each vignette. Covariates included vignette, ASM withdrawal vs continuation, epileptiform EEG, and all their pairwise interactions. We used a logit link, binomial family, and cluster robust standard errors for respondents. Note that the Ferreira‐Atuesta surgical calculator did not include EEG results because some centers contributing data to the model did not have EEG information. Their model also contained one variable that we did not specify in our vignettes (presurgical seizure frequency), because that variable was added to the model after our survey data collection was already in progress. Therefore, in our analyses for surgical vignettes, we averaged across EEG results and displayed sensitivity results for Ferreira‐Atuesta results assuming either monthly or weekly presurgical seizure frequency.

We performed secondary analyses regarding differences between clinician vs calculated predictions. We first assessed more broadly how vignette‐related and respondent‐related covariates influenced differences using generalized linear models. The outcome was the difference between clinician minus Lamberink‐calculated predictions, with a Gaussian link function. We repeated this model using calculated predictions from the Lin model. Additionally, to address the survey's low response rate, we used inverse probability of selection weighting.^28^ Inverse probability of selection weighting seeks to mitigate selection bias by upweighting respondents who had a low predicted probability of participating, to simulate a dataset as if all participants had responded. To do so, first we performed a logistic regression for whether invited AAN members consented to participate. We used independent variables that might influence participation: age, sex, race, region of the USA, academic vs nonacademic practice, percent time clinical vs research, and epileptologist/clinical neurophysiologist. Then, we repeated the main analysis, weighting each respondent by one divided by the predicted probability of consenting to participate.

We then assessed whether viewing calculated predictions changed recommendations (“pre” vs “post” viewing calculated predictions) using scatterplots, violin plots, and bar charts. Recommendations took on values between 0 (extremely unlikely to recommend withdrawal) and 10 (extremely likely). We divided responses by 10 to bound the outcome between 0 and 1 to facilitate using generalized linear models with a logit link. Each recommendation was an outcome, whether the vignette displayed calculated risks was a covariate, and we used cluster‐robust standard errors to account for within‐respondent correlation. Sensitivity models performed inverse probability of selection weighting. We also performed secondary analyses showing scatterplots and lowess curves to assess the degree to which recommendations correlated with risk, and then displayed all results from our generalized linear model describing what respondent and vignette factors influenced recommendations.

## 3 RESULTS

### 3.1 Sample characteristics

We sent the survey to 4923 individuals, of whom 463 consented, 411 passed eligibility questions, and 346 responded to at least one vignette. AAN members consenting to participate were more likely than those not consenting to be White, European, academic, specialized, and researchers (Table 1).

**TABLE 1:** Population description

	Did not consent	Consented	*P*	Include
From AAN data only, to assess selection into the study				
*N*	4036 (92%)	368 (8%)		346^a^
Age, years	50 (41‐58)	51 (42‐60)	0.14	50 (42‐60)
Female	1513/3957 (38%)	147/364 (40%)	0.42	144/332 (43%)
Race				
White	1943/3304 (59%)	220/329 (67%)	<0.01	178/255 (70%)
Black	87/3304 (3%)	14/329 (4%)		11/255 (4%)
Asian	805/3304 (24%)	56/329 (17%)		38/255 (15%)
Region of USA				
Northeast	812/3663 (22%)	63/311 (20%)	0.69	44/240 (18%)
Midwest	722/3663 (20%)	69/311 (22%)		57/240 (24%)
South	1318/3663 (36%)	113/311 (36%)		91/240 (38%)
West	811/3663 (22%)	66/311 (21%)		48/240 (20%)
Cohort				
AAN – US	3687 (91%)	314 (85%)	<0.01	242 (70%)
AAN – Europe	349 (9%)	54 (15%)		46 (13%)
EpiCARE	–	–		58 (17%)
Academic^b^	785/3993 (20%)	111/367 (30%)	<0.01	91/287 (32%)
Percent clinical^b^	90% (75%‐100%)	80% (60%‐95%)	<0.01	80% (60%‐95%)
Percent research	0% (0%‐5%)	0% (0%‐8%)	<0.01	0% (0%‐8%)
Epilepsy/CNP	2043 (51%)	240 (65%)	<0.01	186/288 (65%)
From all data, to describe the respondents				
Academic^b^	–	–		173 (50%)
Percent clinical^b^	–	–		90% (75%‐100%)
% of patients seen for seizures				
<50%	–	–		143 (42%)
~50%	–	–		45 (13%)
>50%	–	–		155 (45%)
% of patients postepilepsy surgery				
None	–	–		115 (33%)
Some	–	–		229 (66%)
All	–	–		2 (1%)
Population				
Mostly ≥18 yrs	–	–		268 (77%)
Board‐certification				
Epilepsy	–	–		143/277 (52%)
CNP	–	–		120/277 (43%)
Specialty				
Epilepsy		–		209/335 (62%)
CNP		–		158/335 (47%)
Years treating patients with epilepsy				
0‐5	–	–		33/277 (12%)
6‐10	–	–		47/277 (17%)
11‐15	–	–		41/277 (15%)
16‐20	–	–		31/277 (11%)
20+	–	–		125/277 (45%)

*Note*: To evaluate for selection bias, we compared AAN member survey recipients who did vs did not consent to complete the survey (“AAN data only”). *P*‐values compare AAN members who did vs did not consent using Chi‐squared and *t*‐tests. For respondents, we then displayed information combining AAN member data plus information they provided during the survey itself to describe our included population.

Abbreviations: AAN, American Academy of Neurology; CNP, clinical neurophysiology.

^a^ These 346 respondents included 242 US AAN members (4001 sent, 314 consented, 287 eligible, 197 completed), 46 European AAN members (403 sent, 54 consented, 49 eligible, 46 completed), and 58 EpiCARE members (519 sent, 95 consented, 75 eligible, 58 completed). Countries represented included the USA (242), Germany (11), Italy (11), Switzerland (8), UK (7), Portugal (6). The remaining all had less than 5: Austria, Belgium, Croatia, Cyprus, Czech Republic, Denmark, Finland, France, Georgia, Hungary, Ireland, Latvia, Lithuania, Luxembourg, Netherlands, Poland, Romania, Russia, Serbia, Slovenia, Spain, Sweden.

^b^ Note variables for academic practice and percent clinical are repeated in the table. The first mention is based only on variables contained in the database of AAN member information that AAN members reported at the time of creating their AAN profile. The second mention of ‘academic’ is whether the individual reported being in an academic practice either through their AAN profile, or else at the time of taking our survey and thus this variable is reported only for the ‘included’ respondents, and hence higher than from AAN profile data alone. The second mention of ‘percent clinical’ is based on responses at the time of taking our survey.

### 3.2 Vignette responses: risk estimation

**FIGURE 1:**
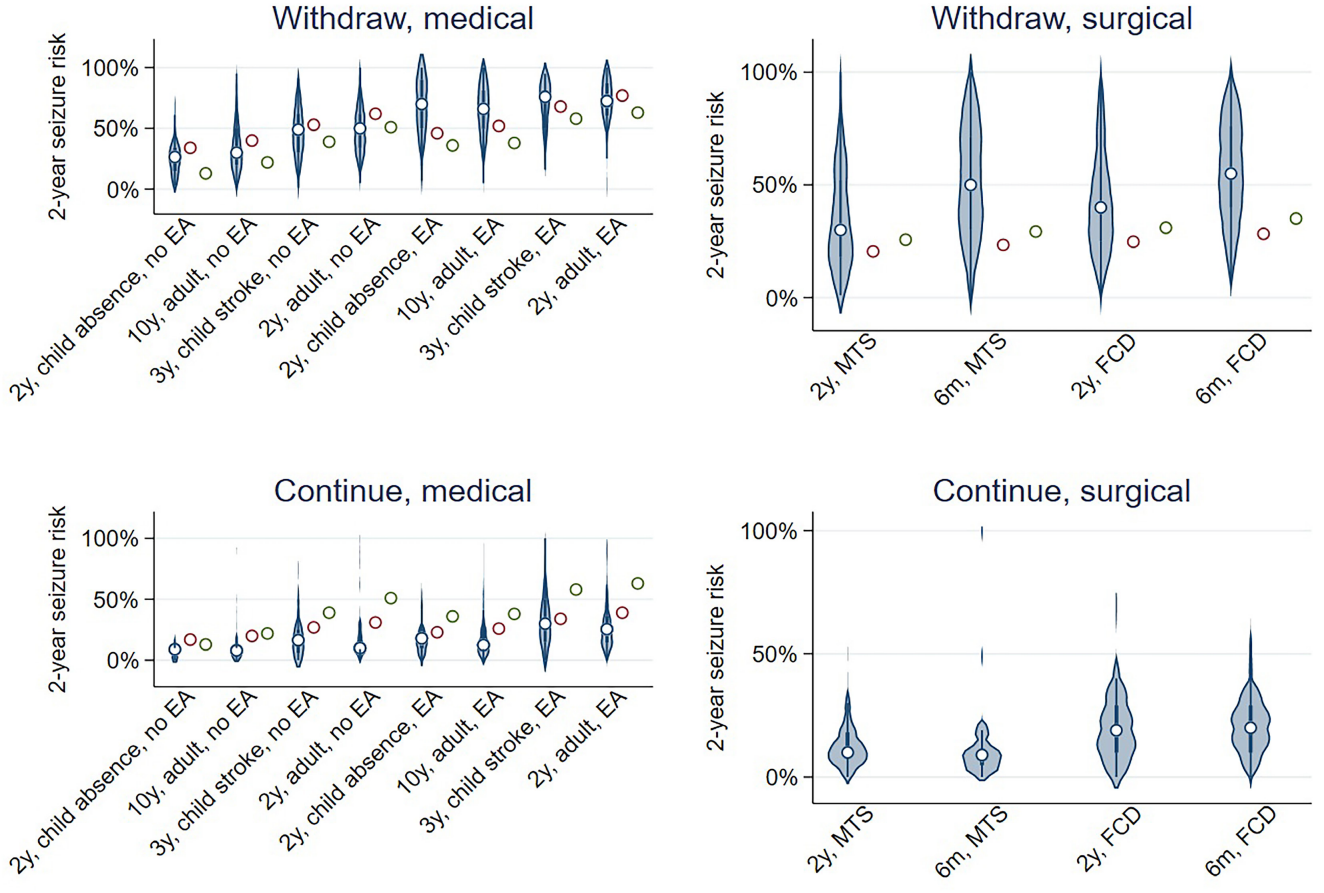
Distribution of clinician vs calculated predictions of 2‐year seizure relapse for each vignette. Left: medical vignettes; Right: surgical vignettes; Top: withdraw ASM; Bottom: continue ASM. Medical vignettes are further separated according to epileptiform EEG abnormalities (left half of medical panels) and no epileptiform EEG abnormalities (right half of medical panels). For medical panels, circles represent calculated predictions from Lamberink (red) or Lin (green) models. For the surgical withdrawal panel, circles represent calculated predictions from Ferreira‐Atuesta assuming a monthly (red) or weekly (green) presurgical seizure frequency. *Interpretation*: There was wide variation in clinical vs calculated predictions of seizure risks throughout vignettes. EA, epileptiform abnormality; FCD, focal cortical dysplasia; MTS, mesial temporal sclerosis

Clinician risk predictions were highly variable across vignettes, often ranging from nearly 0% to 100% (Figure 1; Figure S1). For example, predictions ranged from 5% to 100% for a 2‐year seizure‐free adult without epileptiform abnormalities (5th percentile: 19%; 25th percentile: 34%; 50th percentile: 50%; 75th percentile: 62%; 95th percentile: 86%). Fifty‐eight percent of clinician predictions were within 20% of calculated predictions. Clinician and calculated predictions demonstrated moderate correlation (0.42, 95% CI 38%‐46%).

**FIGURE 2:**
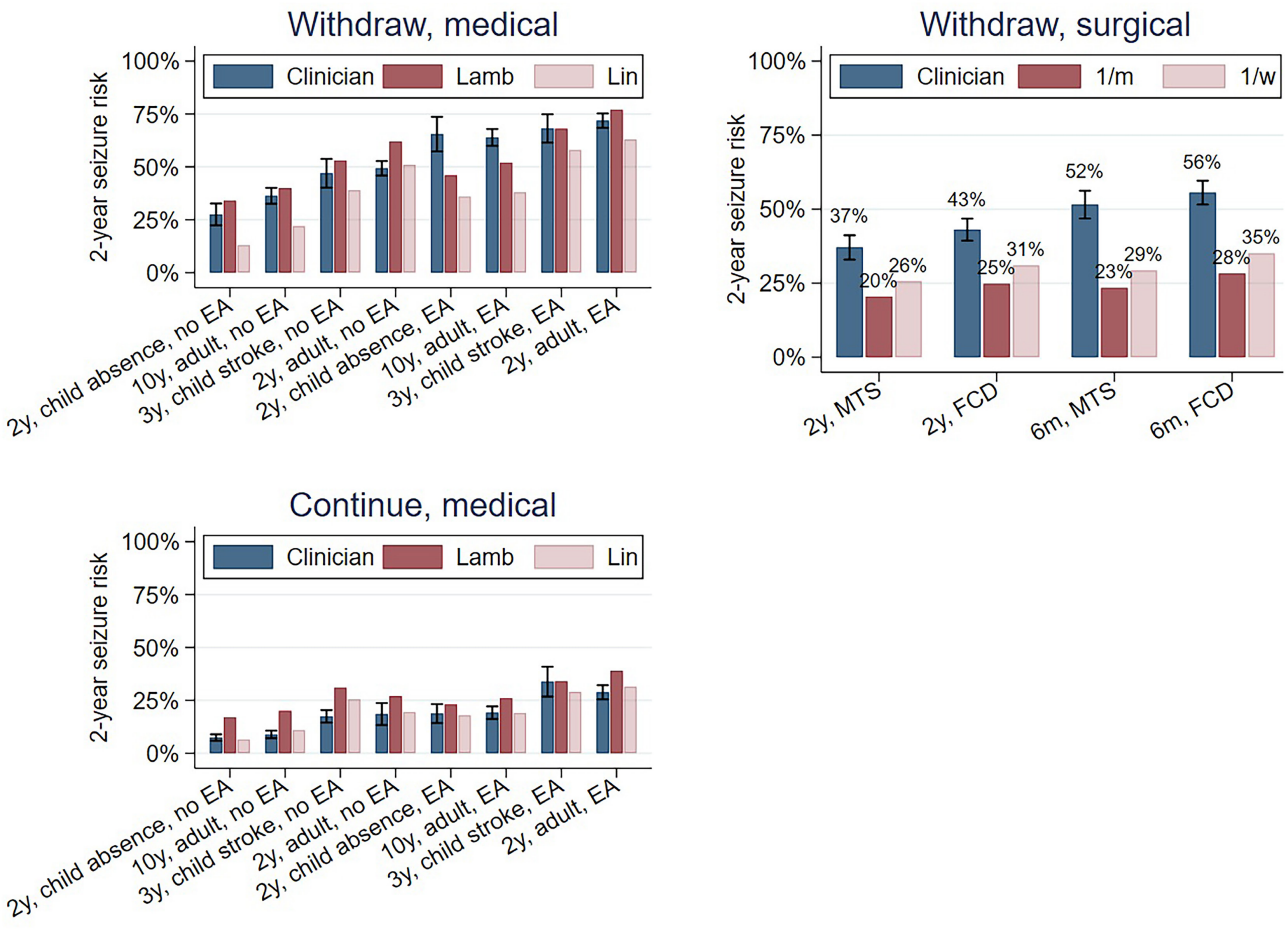
Mean and 95% CIs for clinician vs calculated predictions of 2‐year seizure relapse risk by vignette. *Interpretation*: Mean clinician predictions for medical vignettes were mostly within 10%‐20% of calculated risks. Clinician predictions (“Clinician”) were particularly higher than calculated predictions (“Lamb”: Lamberink et al calculated predictions; “Lin”: Lin et al calculated predictions) for postwithdrawal vignettes with epileptiform EEG findings for the 2‐year seizure‐free child with absence epilepsy and the 10‐year seizure‐free adult. Clinician predictions were higher than calculated predictions for all surgical vignettes, but lower than calculated predictions for continuation medical vignettes without epileptiform EEG abnormalities. Note there is no “Continue, surgical” panel as there was in Figure 1 because randomized data does not yet exist enabling a calculated continuation surgical risk to compare with responses. EA, epileptiform abnormality; FCD, focal cortical dysplasia; MTS, mesial temporal sclerosis

Mean clinician predictions for medical vignettes were almost all within 10%‐20% of calculated predictions (Figure 2). Clinician predictions for postwithdrawal medical vignettes most exceeded calculated predictions for vignettes with epileptiform EEG findings: (a) the 2‐year seizure‐free child with the absence epilepsy (mean clinician prediction 65%, 95% CI 57%‐74%; Lamberink‐calculated prediction 46%, Lin calculated prediction 36%; *P* < 0.05), and (b) the 10‐year seizure‐free adult (mean clinician prediction 64%, 95% CI 60%‐68%; calculated Lamberink prediction 52%, calculated Lin prediction 38%; *P* < 0.05). Clinician predictions tended to be lower than Lamberink‐calculated predictions across medical vignettes without epileptiform EEG findings (*P* < 0.05 for each) and most continuation vignettes.

Clinician predictions for postwithdrawal surgical vignettes all exceeded calculated predictions (eg, focal cortical dysplasia 6‐month seizure‐free mean clinician prediction 56%, 95% CI 52%‐60%; calculated prediction 28%; *P* < 0.05).

Clinicians estimated a greater influence of epileptiform EEGs on postwithdrawal risk (26%, 95% CI 24%‐28%) compared with calculated predictions (14%, 95% CI 13%‐14%). Respondents who indicated they always order EEGs before considering withdrawal estimated an epileptiform EEG to confer greater risk than respondents who do not always order EEGs (Figure S2).

Mean clinician predictions were generally similar across vignettes when comparing epileptologists/clinical neurophysiologists vs all other respondents (Figure S3, which recapitulates Figure 2 except including stratification by specialization). Most respondent characteristics only slightly influenced adjusted differences between clinician and calculated predictions (Figure S4). An exception was that respondents who see only surgical epilepsy patients provided lower clinician predictions, although the CI was wide given only *N* = 16 (compared with 2020 predictions for respondents who see a mixture of medical/surgical patients and 450 predictions for respondents who only see medical patients). Rather, differences were driven by vignette characteristics. Clinician predictions were more likely to exceed calculated predictions for surgical, pediatric, and postwithdrawal cases, and for patients with longer seizure‐free periods or epileptiform EEG findings (*P* < 0.05). Inverse probability of selection weighting yielded similar conclusions (Figure S5).

### 3.3 Vignette responses: recommendations

**FIGURE 3:**
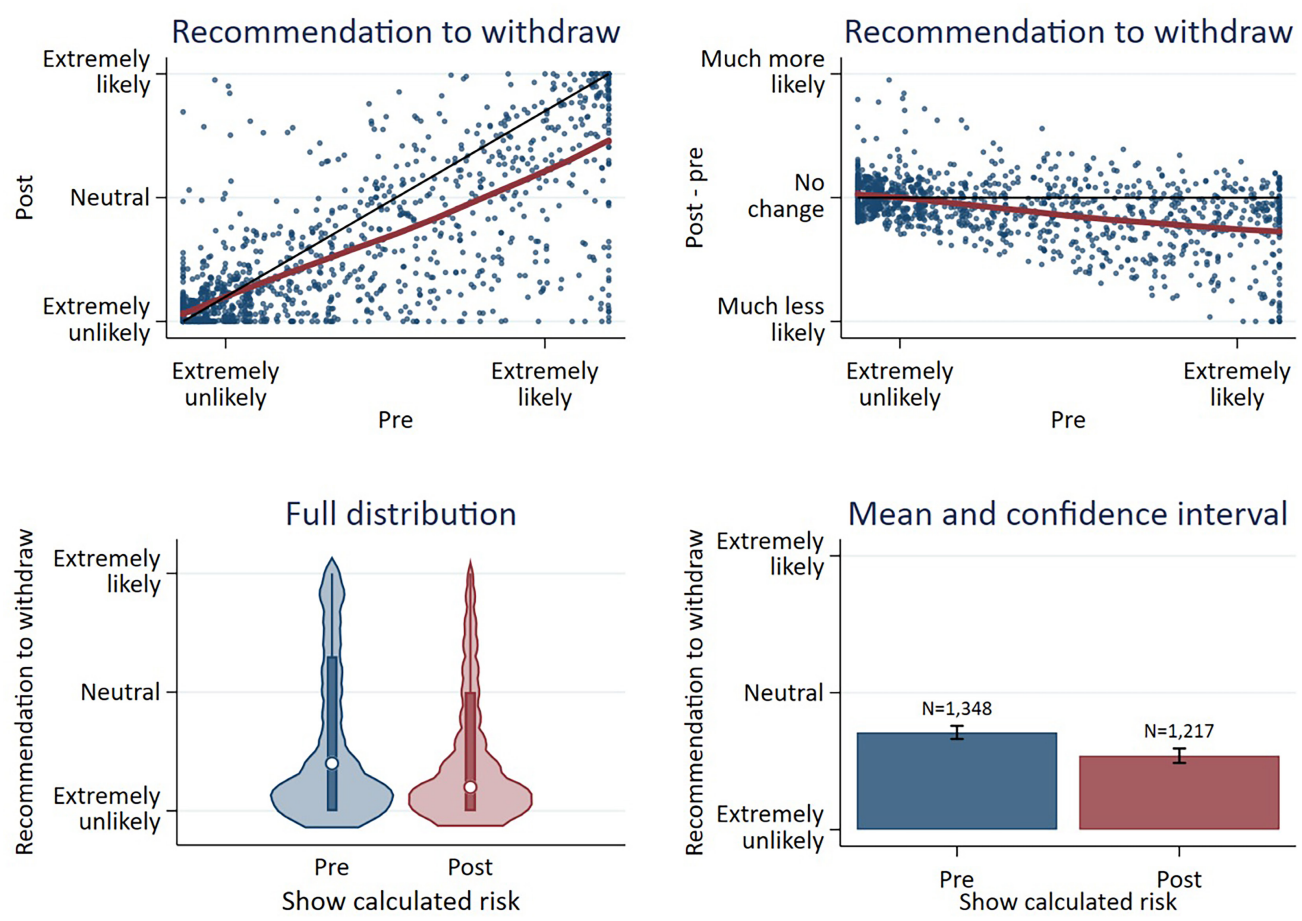
Likelihood to recommend withdrawal before (“pre”) vs after (“post”) viewing results from the calculator for medical vignettes. Upper panels represent scatterplots comparing pre‐ vs postrecommendations (left) or comparing how much recommendations changed after viewing calculated predictions vs “pre” recommendations. Black lines would represent no change, and red curves are loess curves fit to the data. *Interpretation*: Viewing calculator results on average made respondents slightly less likely to recommend withdrawal, particularly for respondents who were more likely to recommend withdrawal at baseline.

**FIGURE 4:**
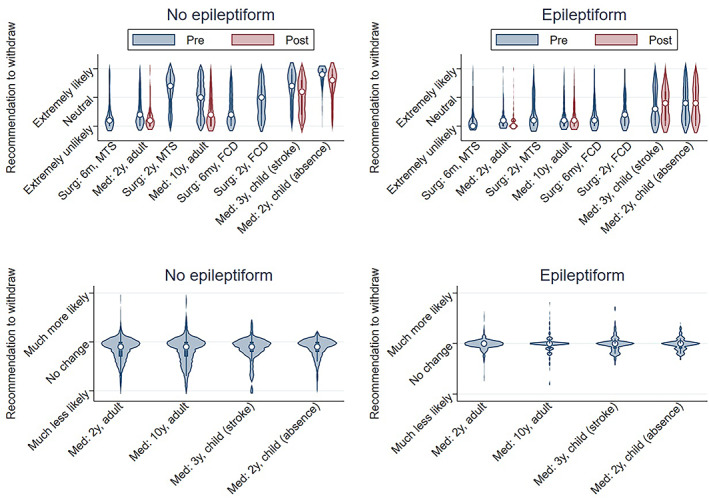
Recommendation to withdraw before (pre) vs after (post) viewing results of the calculator. Top: Distribution of recommendations for each vignette pre‐ vs postviewing results of the calculator (only medical vignettes had a ‘post’ available). Bottom: Distribution of changes in recommendations, post minus pre. *Interpretation*: Variation in recommendations was wide for all vignettes, ranging in many vignettes between 0/10 (extremely unlikely) and 10/10 (extremely likely). On average, viewing calculator results made little difference in recommendations, or else slightly reduced recommendations to withdraw particularly in vignettes without epileptiform EEG abnormalities. Surg: surgical vignette; Med: medical vignette; 6 m: 6 months seizure‐free; 2y: 2 years seizure‐free; 3y: 3 years seizure‐free; 10y: 10 years seizure‐free; MTS: mesial temporal sclerosis; FCD: focal cortical dysplasia

**FIGURE 5:**
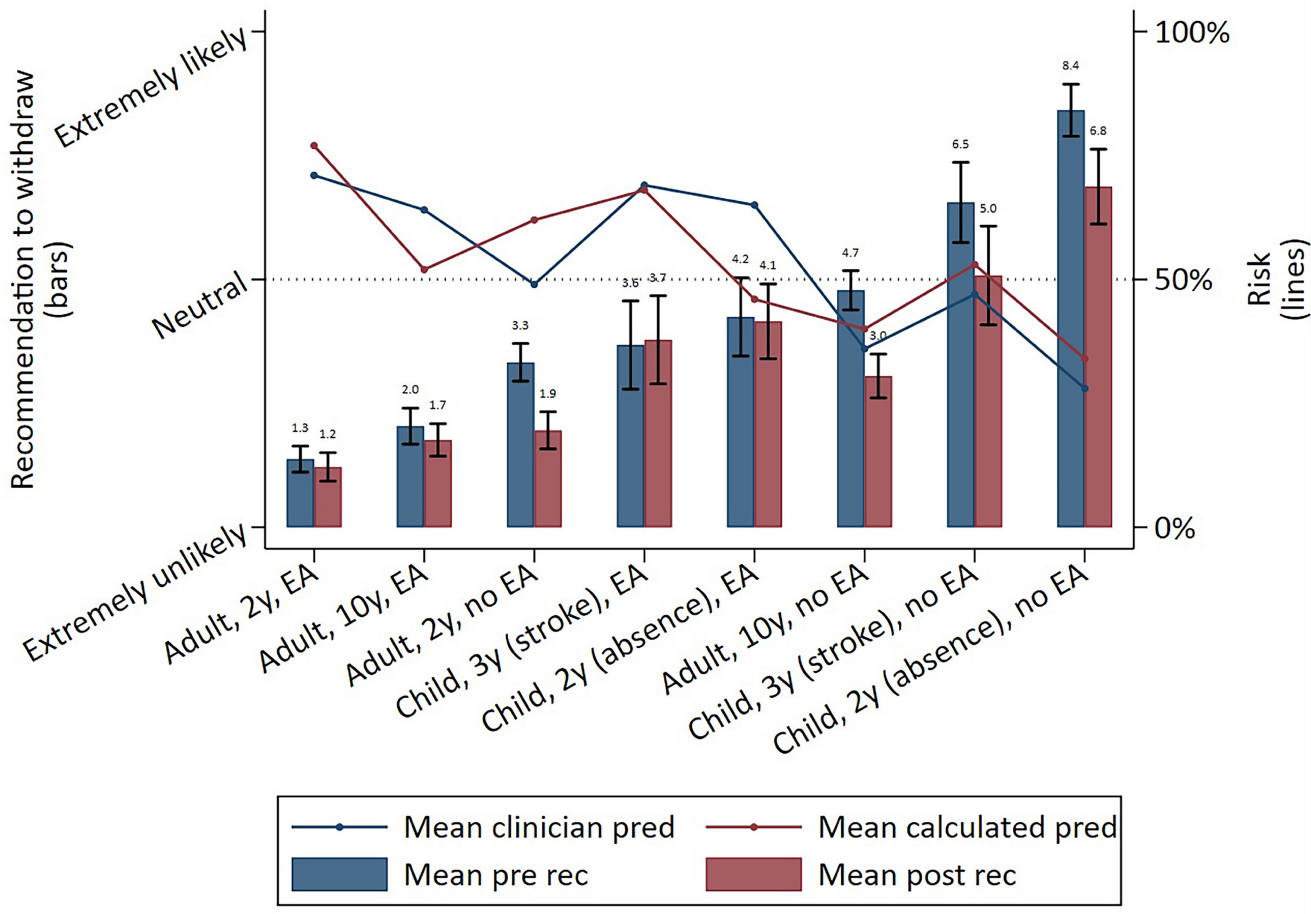
Recommendation to withdraw before (pre) vs after (post) viewing results of the calculator according to vignette, overlaid with postwithdrawal calculated predictions. Bars represent mean (±95% CI) recommendations to withdraw before (blue) vs after (red) seeing calculated predictions and refer to the left y‐axis. Lines represent mean estimations of 2‐year postwithdrawal clinical (blue) and calculated (red) risk predictions and refer to the right y‐axis. *Interpretation*: Viewing calculated risks had no significant effect on recommendations in vignettes with epileptiform EEG abnormalities, but reduced recommendations for vignettes without epileptiform EEG abnormalities. Increasing recommendations to withdraw ASMs corresponded with decreasing vignette risks. 2y, 2 years seizure‐free; 3y, 3 years seizure‐free; 10y, 10 years seizure‐free; EA, epileptiform abnormality; pred, prediction

Viewing calculator results made respondents less likely to recommend withdrawal (Figure 3; pre: mean 3.5/10; post: 2.7/10; mean change −0.8, 95% CI −1.0 to −0.7; *P* < 0.05), particularly for respondents most likely to recommend withdrawal at baseline. Variation in recommendations was wide for all vignettes (Figure 4), often ranging between 0/10 and 10/10. Respondents were least likely to recommend withdrawal for a 2‐year seizure‐free adult with epileptiform EEG abnormalities (mean recommendation before viewing calculator: 1.3/10; mean recommendation after viewing calculator: 1.2/10). Respondents were still unlikely to recommend withdrawal for the 2‐year seizure‐free adult even with a normal EEG (before seeing risk: 3.3; after: 1.9/10), and viewing the calculator most reduced recommendations in cases with a normal EEG (Figure 5). Respondents were the most likely to recommend withdrawal for a child with absence epilepsy and a normal EEG.

In general, recommendations to withdraw increased as the vignette's risk decreased (Figure S6). However, changes in recommendations (after vs before seeing the calculator) correlated only weakly with the degree to which clinicians over‐ vs underpredicted risk compared with calculated predictions (Spearman correlation coefficient 0.31, 95% CI 0.26‐0.37; Figure S7).

Numerous vignette characteristics influenced recommendations, with the strongest recommendation to withdraw for children without epileptiform abnormalities with side effects (8.6/10), and the strongest recommendation against withdrawing for adults with driving needs and no side effects (1.6/10; Figure S8). Epileptologists/clinical neurophysiologists were more likely to withdraw both before and after viewing calculated predictions compared with nonspecialists for several vignettes, particularly for childhood cases (Figure S9). After adjusting for all respondent and vignette characteristics, the strongest factor influencing recommendations was whether respondents treat only surgical patients (Figures S10 and S11), which predicted higher likelihood for recommending withdrawal (8.6/10, 95% CI 8.0/10‐9.2/10; vs respondents who treat some surgical patients 3.2/10, 95% CI 3.0/10‐3.5/10; *P* < 0.05). Although, there were only 10 datapoints from surgical‐only respondents, of the total 3181 datapoints in this regression. Vignette characteristics increasing the likelihood of recommending withdrawal were pediatric or surgical vignettes, side effects, normal EEG, longer seizure‐free period, not seeing the calculator, and the absence of a lesion (*P* < 0.05). For example, an epileptiform EEG reduced recommendations on average by 1.9 (95% CI 1.7 to 2.1) points on a 10‐point scale.

### 3.4 Frequency/barriers of using calculators

Of 279 respondents answering whether they use the Lamberink online risk calculator, responses were: 151 (54%) never, 66 (24%) less than half the time, 19 (7%) half the time, 19 (7%) more than half the time, and 24 (9%) always. Of the 128 respondents who ever use the calculator, respondents find it: 20 (16%) very useful, 29 (23%) useful, 41 (32%) somewhat useful, 21 (16%) a little useful, 1 (1%) not at all useful, and 16 (13%) “I don't know.” Of the 280 responding about barriers to using the calculator, top barriers included being unsure regarding its accuracy (122; 44%), not integrated into the medical record (116; 41%), unsure if it applies to a given patient (112; 40%), unaware that such a calculator existed (103; 37%), unsure how to find it (89; 32%), not enough time to use it during visits (61; 22%), and 14 (5%) indicated there are no barriers. Free‐text comments included the importance of patient preferences, that calculated risk may not influence their decisions unless calculations were very close to 0% or 100%, and difficulty for patients thinking in terms of chance rather than certainty.

## 4 DISCUSSION

We performed an international survey exploring how US and European neurologists estimate seizure risk and make recommendations about ASM withdrawal in patients with well‐controlled epilepsy. While mean clinician predictions were mostly close to calculated predictions, we found wide variation in clinician predictions and recommendations within all vignettes. Respondents overestimated risk in the lowest‐risk vignettes with epileptiform abnormalities (ie, childhood absence epilepsy; 10‐year seizure‐free adult; surgical patients) and overestimated the influence of EEG abnormalities compared with best‐available calculation. Viewing calculator results reduced recommendations to withdraw for vignettes with no epileptiform abnormalities but made little difference in recommendations for vignettes with epileptiform abnormalities.

Variation in recommendations is not unexpected. Current science does not inform any single optimal risk cutoff, all clinicians may have different thresholds tailored to their population, and prior work has suggested that epileptologists might be more likely to withdraw ASMs perhaps related to greater comfort with such decisions.^29^ However, wide variation in risk estimation when shown the same clinical data is concerning, given two clinicians with the same risk threshold seeing similar patients may reach very different conclusions. This encourages continuing to develop accurate seizure risk prediction tools to better standardize risk estimation. The existing calculator for medical patients had modest discrimination during development (area under the curve 0.65) in addition to variable results from three external validation studies including one with overpredictions,^25^ one with acceptable calibration,^24^ and one with poor calibration.^26^ Given the best currently available prediction tools have shown moderate performance, wide variation in clinician predictions is understandable. Also, one may have hypothesized “more accurate” risk estimation in clinicians with greater subspecialization or years of experience, but this was not the case. Rather, our data suggested that risk estimation may not align with best‐available risk calculators particularly for certain types of patients, rather than for certain provider types.

Our results may question certain common practices regarding ASM withdrawal. For example, two‐thirds of respondents always obtain EEGs before withdrawal. However, although clinicians estimated that epileptiform EEG abnormalities increased adjusted risk by 30%‐40% and epileptiform EEG abnormalities had a modest influence on recommendations, calculated predictions suggested that epileptiform EEG abnormalities increased risk by only approximately 10%‐15%. Thus, always obtaining an EEG may not be required, depending on what absolute risk difference a clinician feels is clinically meaningful. Several free‐text responses expressed the importance of EEGs yet also underscored that some clinicians would never advise withdrawal until seizure risk dropped very close to 0%, which is not achievable even with a normal EEG, even for patients who have never had a seizure.^30^ It is, however, difficult to extrapolate from our data firm recommendations regarding when to order an EEG, as this remains a highly individualized decision. Ideally this decision would be made by first articulating what posttest seizure risk probability might change decisions, compared with an accurate understanding of whether EEG findings would shift the posttest probability of subsequent seizures sufficiently to cross such a threshold. The most updated guidelines also express considerable uncertainty regarding optimal EEG durations or utility of sleep deprivation in this setting, and even whether EEG findings affect risk in adults.^12^ Thus, clear future research gaps remain in our understanding of optimal EEG ordering practices. At least, what we can say from our data is that EEG findings influenced decision noticeably more before viewing calculated predictions compared with after viewing calculated predictions. This suggests that having a more realistic understanding of the influence of EEG findings on seizure risk could lead to less reliance upon EEG data in making withdrawal decisions.

Furthermore, our results suggest that clinicians may be too cautious regarding postsurgical ASM withdrawal. Calculated predictions for postoperative vignettes were about 20%‐30% lower than clinician predictions, often about 50% lower than clinician predictions. Furthermore, calculated predictions suggested that awaiting 2 years compared with 6 months of seizure‐freedom reduced the patient's risk only by about 3%. Thus, for a postoperative patient who plans to eventually withdraw ASMs, early withdrawal may be justified, despite average recommendations being in the range of “extremely unlikely” to “unlikely” for our surgical cases. The TimeToStop study in children showed that ASM withdrawal as soon as 6 months after epilepsy surgery does not influence long‐term seizure outcome,^31^ although it is not known whether this also applies to adults.

Viewing calculated risk particularly reduced recommendations without epileptiform EEG abnormalities. For example, the mean clinician‐predicted postwithdrawal risk was nearly identical to the Lamberink‐calculated risk for a 10‐year seizure‐free adult. Nonetheless, viewing calculated risk still reduced recommendations to withdraw. Perhaps making decisions according to clinician's intuitive understanding that this is a relatively low‐risk case may lead to more liberal recommendations, whereas requiring clinicians to articulate probabilities may lead to more conservative recommendations.

Almost half of respondents ever use the available online risk calculator, and 40% reported being unsure whether it is accurate. We recognize that no there is no single “gold‐standard” model, which is why we took caution to present numerous methods to calculate risk for each case. Developing a model with high accuracy and widespread applicability represents a key, challenging, research goal. Even if a calculator predicted risk perfectly, other implementation barriers still include dissemination and integration into clinicians' workflow, deciding whether and how to communicate complex probabilities to patients who may have low numeracy,^32^ and most fundamentally, understanding what probability threshold, if any, merits ASM withdrawal.

Our work has limitations. The response rate was low. Given Table 1, we could have overrepresent academic, specialist, researchers. While we invited a large, diverse audience, and an international survey is the optimal study design to address our research questions, high response rates are difficult to achieve during physician research.^33^ However, because we anticipated this potential pitfall, we specifically used AAN data because AAN contains data regarding all its members, including those who did not participate in this survey. Knowing demographics and professional descriptors of both responders and nonresponders enabled us to leverage a key technique toward quantitative bias assessment—inverse probability of selection weighting. This technique reweights individuals in a sample according to the probability that they ended up participating, to be more representative of the source population. This directly addresses the possibility of a “missing at random” missing data mechanism (ie, missing values explained by observed variables), to the degree that we captured the most important drivers influencing whether invitees chose to participate. We believe that we have captured the most important drivers, such as subspecialization, academic setting, research effort, geography, age, race, and sex. We acknowledge, however, that residual nonresponse bias (“missing not at random”) remains possible, and it is impossible to capture or know the impact of all factors influencing both the intended outcomes and whether invited individuals participated. Nonetheless, it was encouraging that after accounting for the probability of participation, there were no meaningful differences to our conclusions. Given the major source of missingness stemmed from deciding to participate in the study rather than missing data after deciding to participate in our study, we focused our analytic efforts to address bias on factors influencing the consenting process, rather than imputation of missing responses after consenting.

There were several other limitations. We did not survey nonneurologists, who also provide care for this population.^19^ We also did not allow the option of reducing doses or switching ASMs when we presented vignettes. Our questions mimicked existing guidelines on this topic which present withdrawal as a dichotomous decision. Adding considerations of reducing or switching ASMs into our survey would have added further considerable complexity and length. And as stated above, only so many vignettes are feasible within any survey. For example, we did not include pediatric surgical cases.

## 5 CONCLUSIONS

Respondents provided highly variable clinical risk predictions and recommendations for ASM withdrawal. Wide variation encourages efforts at developing more evidence‐based approaches to determining which patients benefit from continued ASM treatment vs withdrawal and future efforts developing point‐of‐care seizure risk prediction tools integrated into the electronic medical record. Clinicians overestimated the influence of epileptiform abnormalities, seizure‐free duration, and withdrawal risk for surgical patients compared with calculated results, which may question widespread ordering of EEGs or time‐based seizure‐free thresholds and may encourage using calculated predictions in such scenarios. Viewing calculator results reduced recommendations to withdraw particularly for cases with a normal EEG. Future research is needed regarding how low seizure risk should be before ASM withdrawal.

## AUTHOR CONTRIBUTIONS

S.W.T. conceived of and designed the study, executed the statistical analysis, and wrote the manuscript. C.E. Rheaume collected the data. All authors assisted with designing the survey, interpretation, and manuscript editing.

## FUNDING INFORMATION

These funding sources had no role in the survey, other than American Academy of Neurology which funded staff to execute this survey. Dr Terman is supported by the American Epilepsy Society Susan S Spencer Clinical Research Training Scholarship and the Michigan Institute for Clinical and Health Research J Award UL1TR002240. Dr Terman was a member of the Junior Investigator Intensive Program of the US Deprescribing Research Network, which is funded by the National Institute on Aging (R24AG064025). Ms van Griethuysen is supported by the friends UMC Utrecht/MING Fund. Ms Rheaume is employed by the American Academy of Neurology. Dr Slinger is supported by the friends UMC Utrecht/MING Fund. Ms Haque reports no relevant funding. Dr Smith reports no relevant funding. Dr Kerr is supported by National Institutes of Health R25NS065723, U24NS107158, and the American Epilepsy Society. Dr van Asch reports no relevant funding. Dr Otte is supported by the friends UMC Utrecht/MING Fund. Dr Ferreira‐Atuesta reports no relevant funding. Dr Galovic reports no relevant funding. Dr Burke is supported by National Institutes of Health National Institute of Aging R01 AG068410. Dr Braun is supported by the friends UMC Utrecht/MING Fund.

## CONFLICT OF INTEREST

The authors report no financial disclosures or competing interests relevant to the study. C.E. Rheaume is employed by the American Academy of Neurology Member Insights team who executed this survey.

## ETHICAL APPROVAL

We confirm that we have read the Journal's position on issues involved in ethical publication and affirm that this report is consistent with those guidelines.

## Supporting information


Figure S1

Figure S2

Figure S3

Figure S4

Figure S5

Figure S6

Figure S7

Figure S8

Figure S9

Figure S10

Figure S11

Appendix S1

Table S1

## Data Availability

The American Academy of Neurology is the sole owner of the raw data.
