# Supplementary figures and images for: Antiseizure medication withdrawal risk estimation and recommendations: A survey of American Academy of Neurology and EpiCARE members
Source: Epilepsia Open. 2023 Feb 14;8(2):386–98. doi: 10.1002/epi4.12696 (PMC10235556; doi:10.1002/epi4.12696)

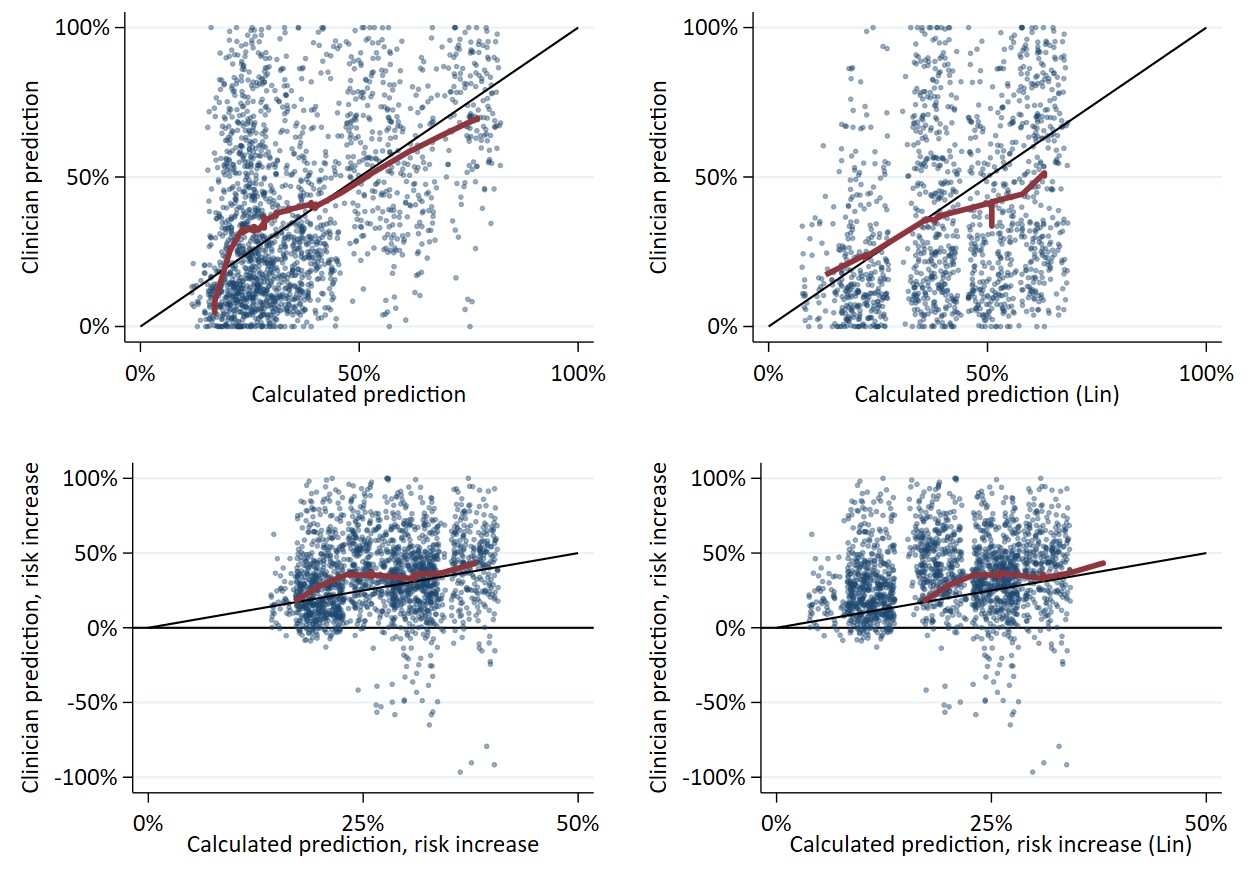

Supplement: Supplementary file 1 — Figure S1 [file EPI4-8-386-s011.jpg]

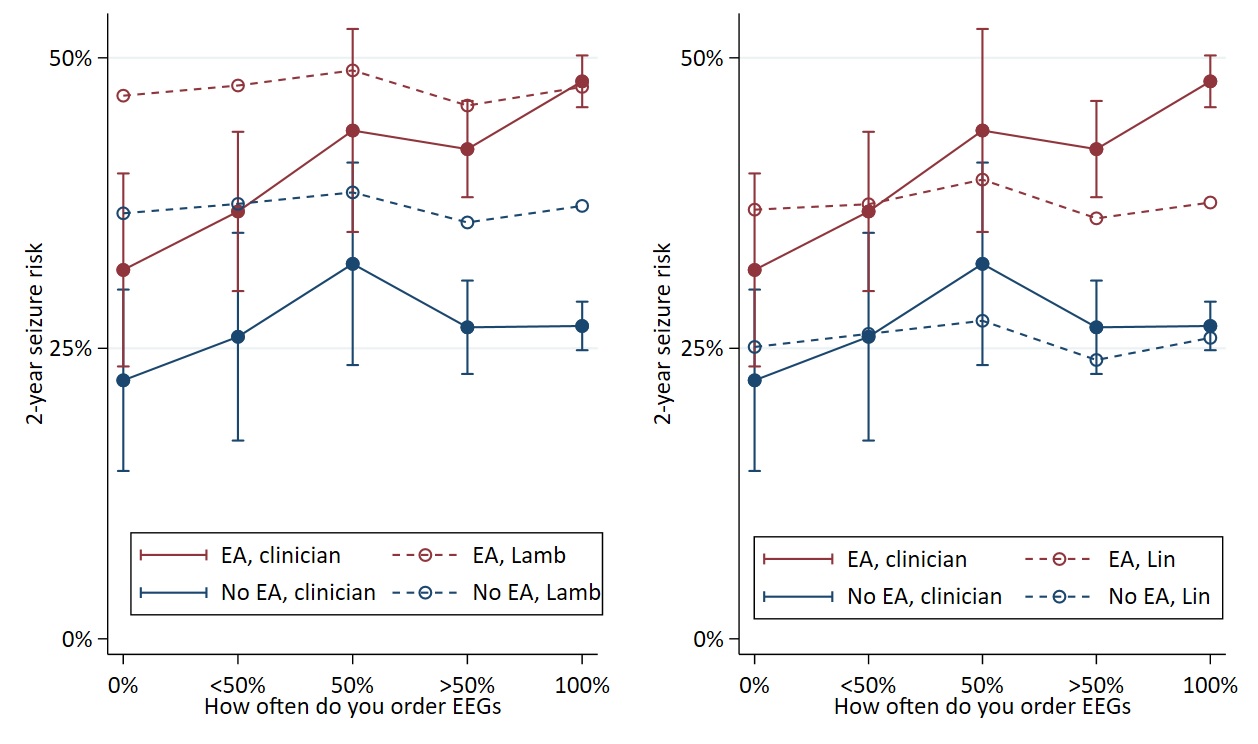

Supplement: Supplementary file 2 — Figure S2 [file EPI4-8-386-s005.jpg]

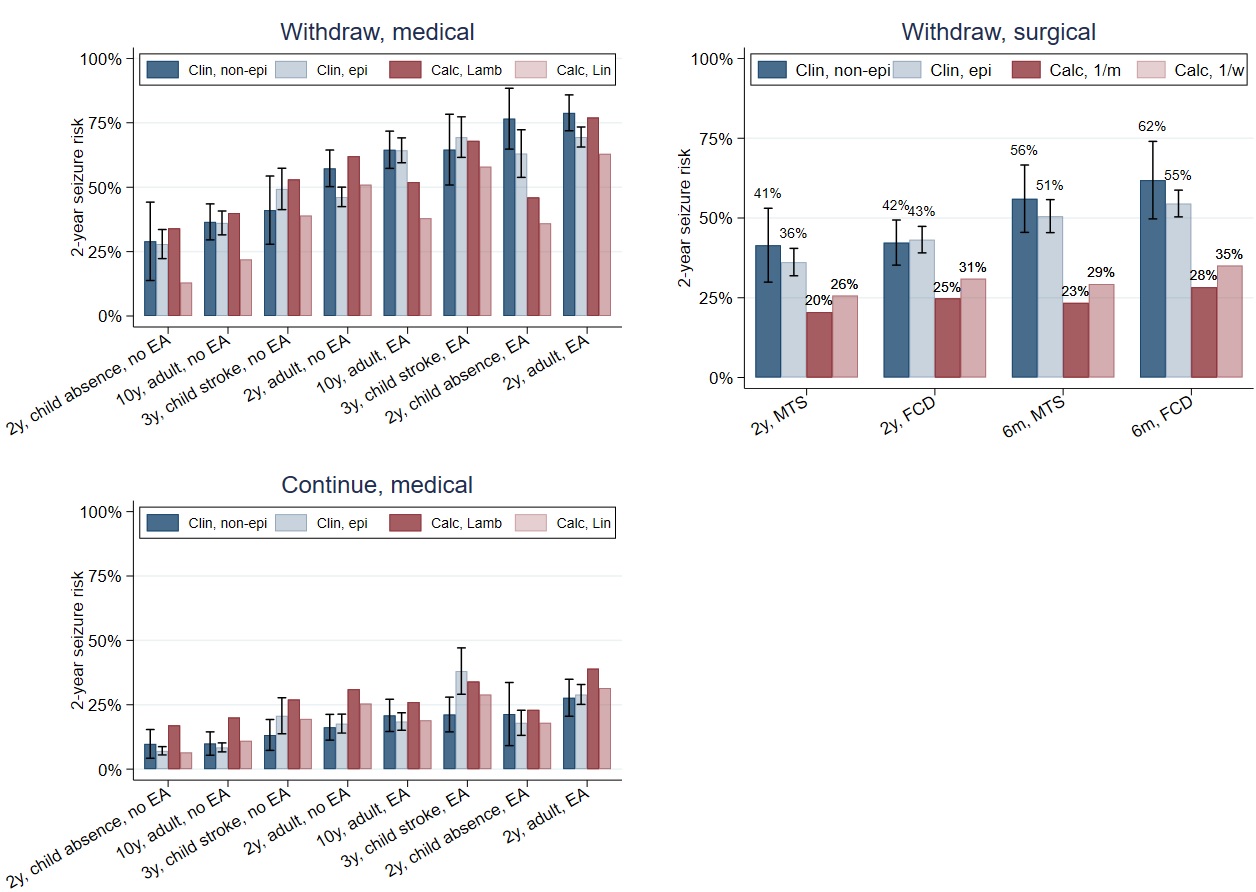

Supplement: Supplementary file 3 — Figure S3 [file EPI4-8-386-s003.jpg]

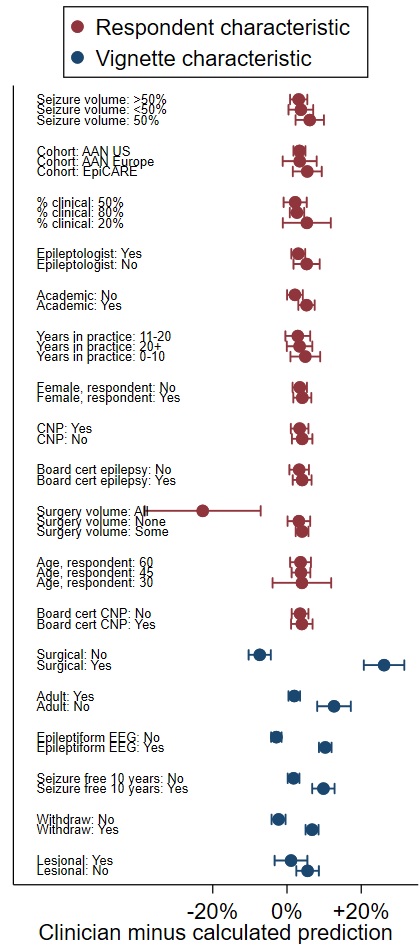

Supplement: Supplementary file 4 — Figure S4 [file EPI4-8-386-s008.jpg]

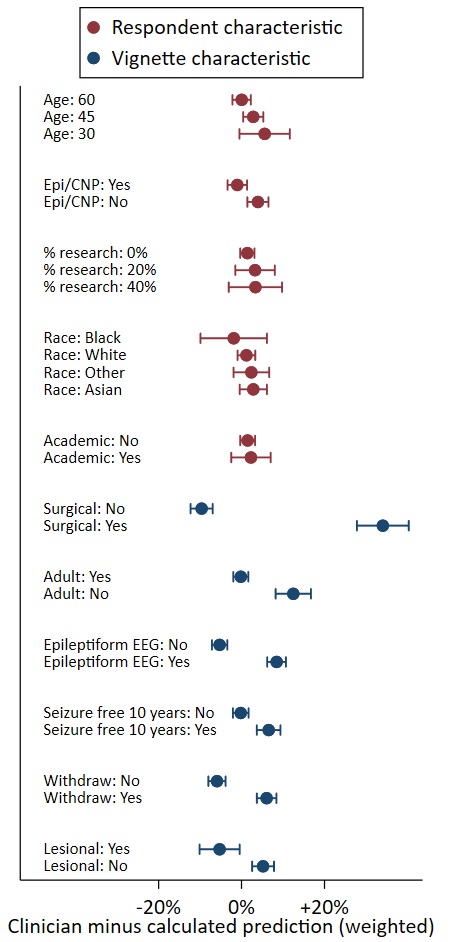

Supplement: Supplementary file 5 — Figure S5 [file EPI4-8-386-s012.jpg]

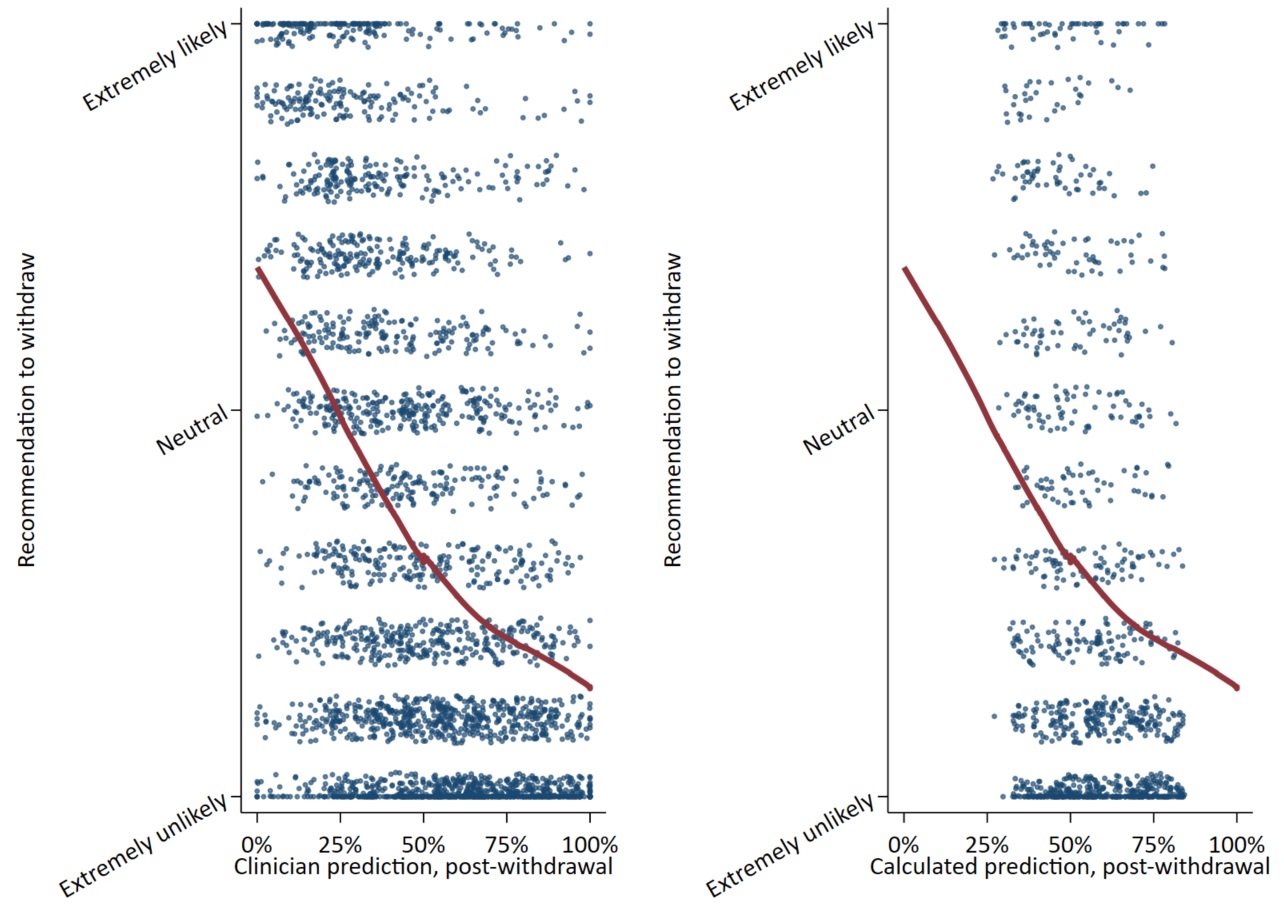

Supplement: Supplementary file 6 — Figure S6 [file EPI4-8-386-s013.jpg]

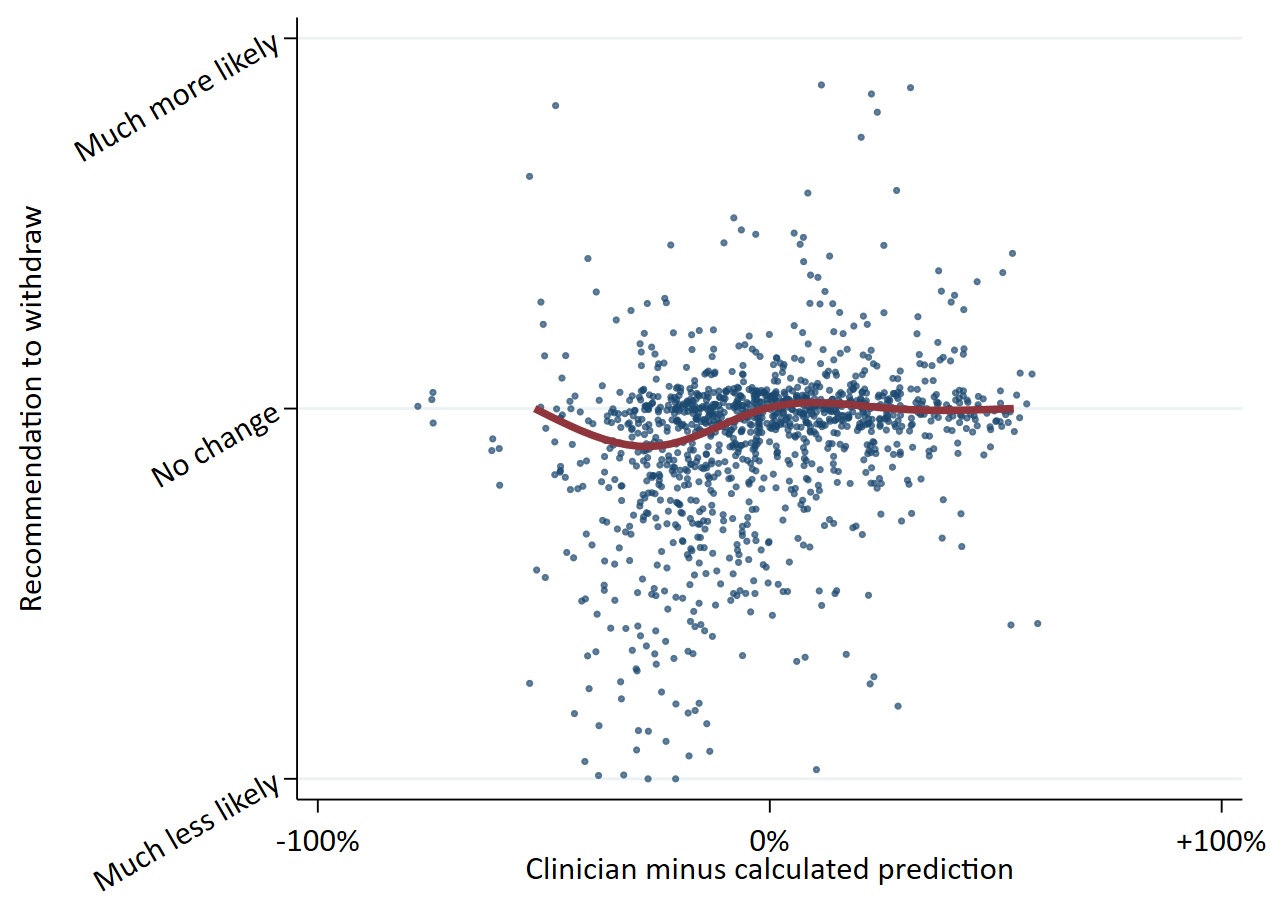

Supplement: Supplementary file 7 — Figure S7 [file EPI4-8-386-s001.jpg]

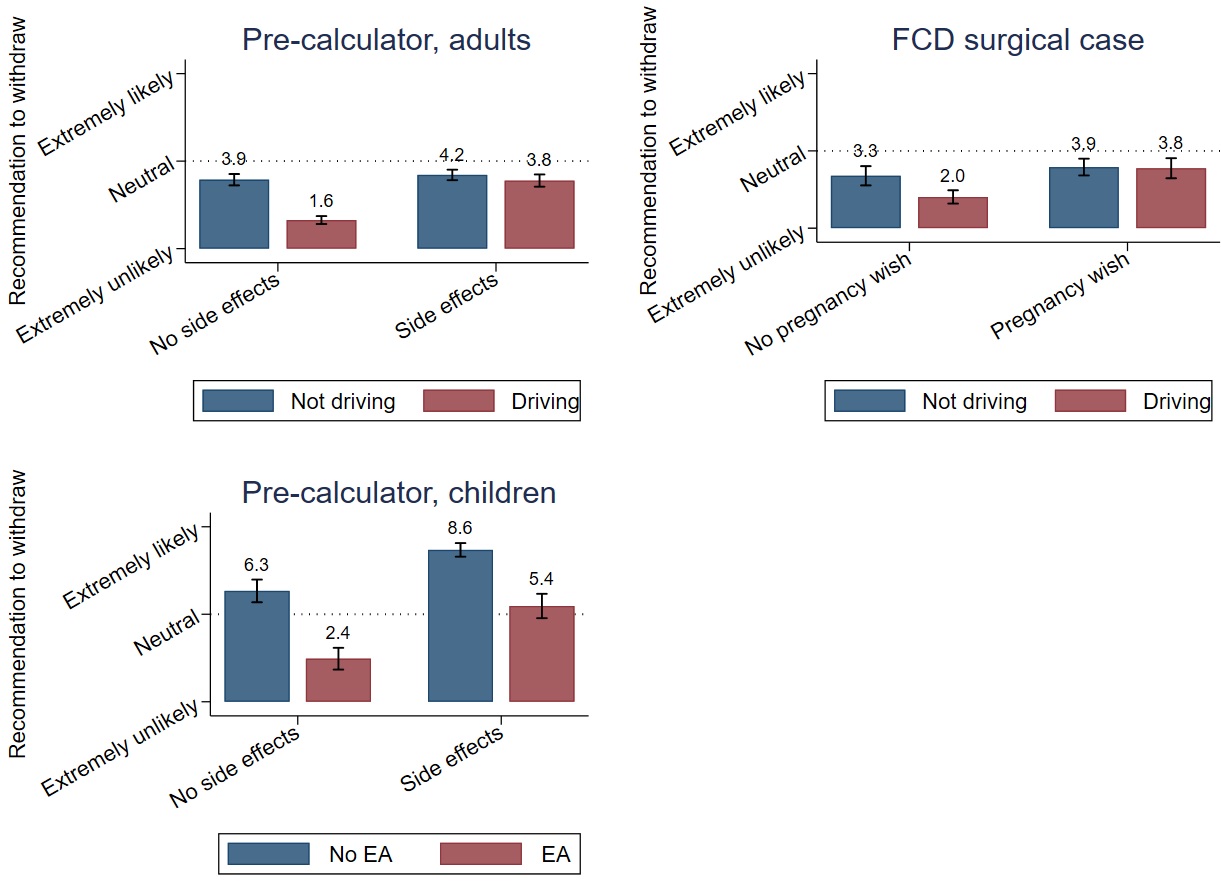

Supplement: Supplementary file 8 — Figure S8 [file EPI4-8-386-s004.jpg]

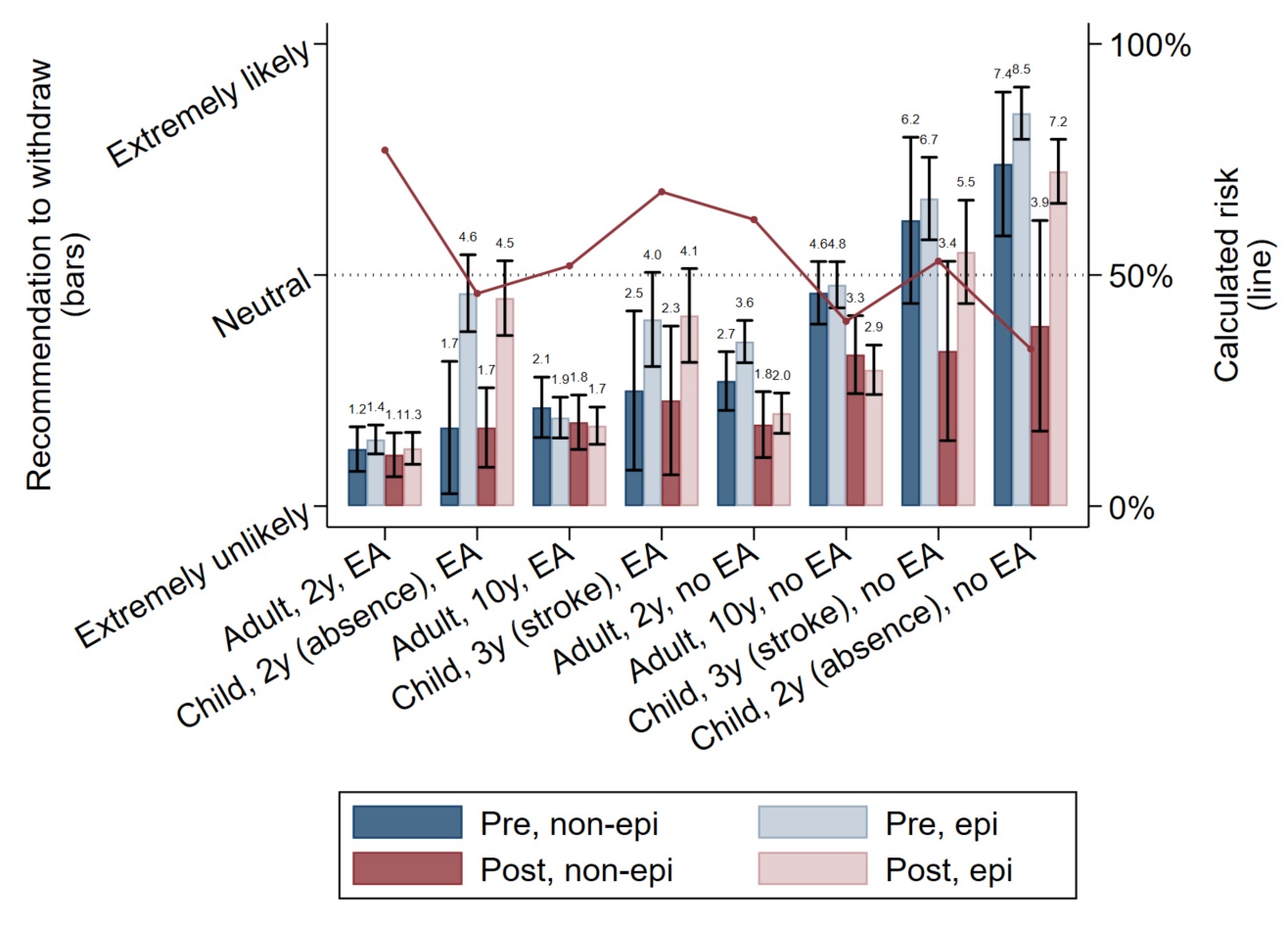

Supplement: Supplementary file 9 — Figure S9 [file EPI4-8-386-s007.jpg]

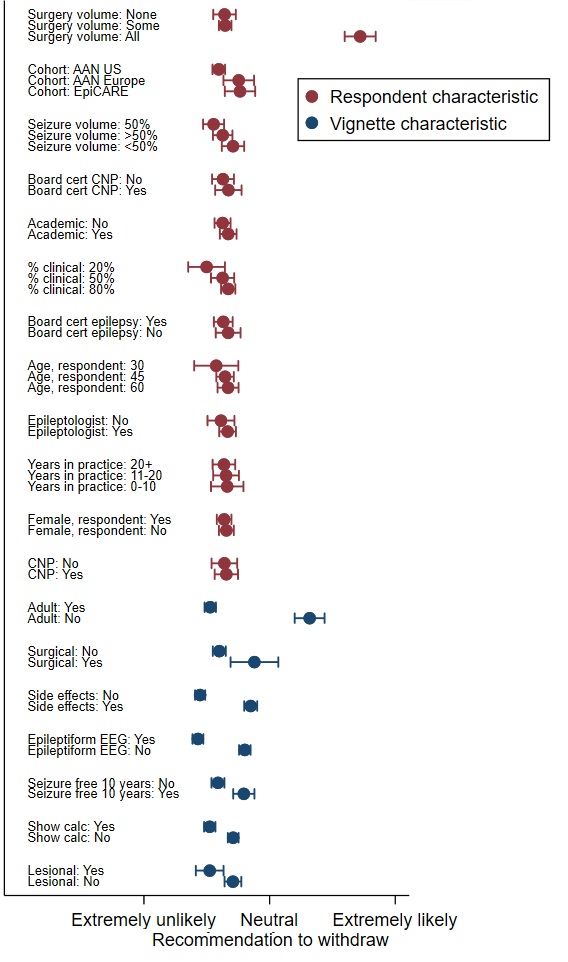

Supplement: Supplementary file 10 — Figure S10 [file EPI4-8-386-s010.jpg]

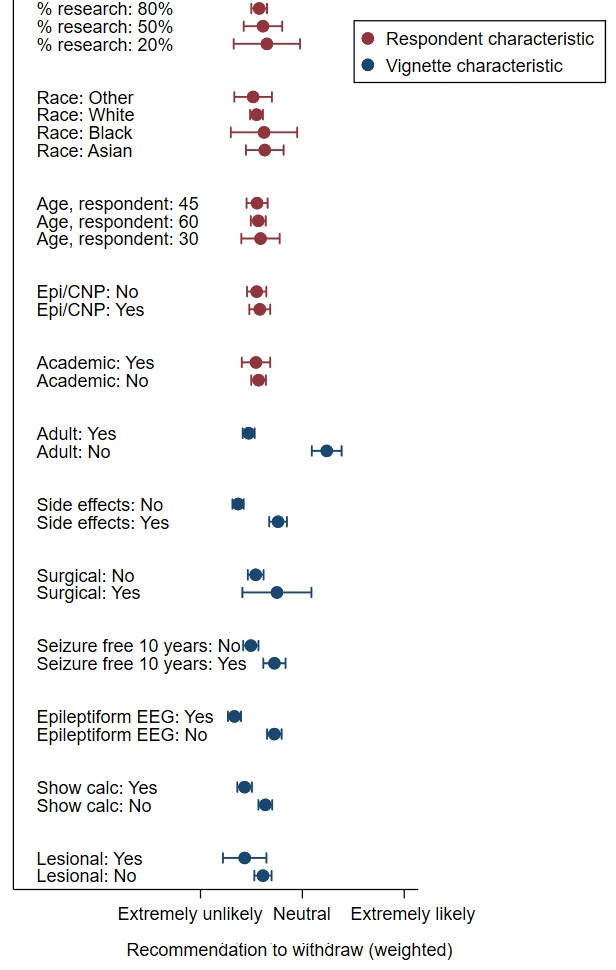

Supplement: Supplementary file 11 — Figure S11 [file EPI4-8-386-s002.jpg]
