# Supplementary material for: Antiseizure medication withdrawal risk estimation and recommendations: A survey of American Academy of Neurology and EpiCARE members
Source: Epilepsia Open. 2023 Feb 14;8(2):386–98. doi: 10.1002/epi4.12696 (PMC10235556; doi:10.1002/epi4.12696)
Supplement: Supplementary file 13 — Table S1 [file EPI4-8-386-s009.docx]

**Supplemental Table 1:** Vignettes.

| **Vignette** | **Adult** | **Adult** | **Surgical, MTS** | **Surgical, MTS** | **Surgical, FCD** | **Surgical, FCD** | **Child, absence** | **Child, stroke** |
| --- | --- | --- | --- | --- | --- | --- | --- | --- |
| **Sz-free** | 2y | 10y | 6m | 2y | 6m | 2y | 2y | 3y |
| **Age, onset** | 26 | 26 | 15 | 15 | 15 | 15 | 8 | 5 |
| **Age, last sz** | 28 | 28 | 30 | 30 | 30 | 30 | 9 | 8 |
| **Age, current** | 30 | 38 | 30.5 | 32 | 30.5 | 32 | 11 | 11 |
| **Sex** | M | M | M | M | F | F | F | F |
| **Duration, sz’s, y** | 2 | 2 | 15 | 15 | 15 | 15 | 1 | 3 |
| **Duration, sz-free, y** | 2 | 10 | 0.5 | 2 | 0.5 | 2 | 2 | 3 |
| **# seizures** | 6 | 6 | Innum | Innum | Innum | Innum | Innum | 6 |
| **Semiology** | FBTC | FBTC | FBTC | FBTC | FBTC | FBTC | Abs.+GTCs | FM |
| **Etiology** | UK | UK | MTS | MTS | FCD | FCD | UK | Stroke |
| **Surgical** | N | N | Y | Y | Y | Y | N | N |
| **FH** | N | N | N | N | N | N | N | N |
| **FS** | Y | Y | Y | Y | N | N | N | N |
| **DD** | N | N | N | N | Y | Y | N | Y |
| **ASM** | Lev | Lev | Lev | Lev | Phen | Phen | VPA | Lev |
| **# responded** | 136 | 130 | 73 | 73 | 76 | 76 | 40 | 38 |

Sz-free: duration since last seizure; 2y: 2 years seizure-free; 10y: 10 years seizure-free; 6m: 6 months seizure-free; sz: seizure; Innum: innumerable; FBTC: focal to bilateral tonic-clonic; Abs: absence; GTCs: generalized tonic-clonic seizures; FM: focal motor; MTS: mesial temporal sclerosis; FCD: focal cortical dysplasia; UK: unknown; FH: family history; FS: febrile seizures; DD: developmental delay; ASM: antiseizure medication; Lev: levetiracetam; Phen: phenytoin; VPA: valproic acid; # responded: number of neurologists who provided at least one clinician prediction or recommendation for each vignette
